# American Academy of Dental Science

**Published:** 1897-11

**Authors:** 

**Affiliations:** American Academy of Dental Science


### AMERICAN ACADEMY OF DENTAL SCIENCE.

The regular monthly meeting of the American Academy of Dental Science was held at Young's Hotel, Boston, March 3, 1897, at six o'clock.

The paper for the evening was read by Gardner T. Swarts, M.D. Subject, "Diphtheria."

(For Dr. Swarts's paper, see page 705.)

### DISCUSSION.

*Chairman Cooke.*—I am sure we have all enjoyed the interesting talk which our brother has given us, and the subject is now open for discussion by the members. If there are any points not fully understood by the members, I am sure Dr. Swarts will be pleased to answer any questions.

*Dr. Werner.*—I would like a repetition of the last part of his address, where he spoke of the disinfecting method that is in use now. I do not think I quite understood it.

*Dr. Swarts.*—Let us take, for instance, a tin,—any ordinary tin, such as a pan for setting out milk,—pack it with asbestos, and pour in some ordinary wood alcohol and ignite it, and the fumes coming up are caught by means of a circular tin which is made to fit over the base, and resembles somewhat a stove-pipe. About half-way up this circular tin let us insert a diaphragm of asbestos, which has little holes in it. That asbestos has been platinized, and is in such a condition that it can be brought up to a white heat without doing it any injury. Now, after that diaphragm has been heated, the alcohol flame is extinguished by placing a cover over the top of the circular tin for a moment; then the vapor of the alcohol in passing through the heated diaphragm becomes formaldehyde. It is wood alcohol up to the heated diaphragm, beyond that it is formaldehyde gas. The value of this gas as a disinfectant has been known for some time, but it has not been possible for us to make practical use of the knowledge, on account of the great expense attending the manufacture of it, the cost of the solutions for its production previous to the perfection of this simple apparatus which I have explained being something like two to three dollars a pound. It is very effectual. You may place some of these diphtheria bacilli, or any other organisms, in any part of a room,—under the carpet, in the bed, in the closets, or anywhere you choose,—and when the formaldehyde is generated in that room it will be impossible to protect them from it. It will penetrate any kind of cloth, as well as the wall-paper, and even wood. In whatever part of the room the organisms may have lodged, it will find them and destroy them.

*Dr. Werner.*—Does it do any harm to the room or anything in it?

*Dr. Swarts.*—None whatever. It is a dry gas which apparently has no effect on any substance in the room.

Of course, the point we have been after most in this question of disinfection is the destruction of the tubercle bacillus. You know that the railroads carry a great many people West and South who are in different stages of consumption. The Pullman cars in which these patients ride are thoroughly cleaned every time they come in, say once a week, but, of course, it would be impracticable to use the ordinary method of fumigating. The method which is commonly used by them is to force a powerful blast of air through the upholstery and cushions, by which means the dust is freely blown out of the seats or the corners of the car in which it may have accumulated. They have to use very powerful air-pumps, and you will naturally see that the railroads have spent a great deal of money in trying to find something to kill these organisms without injury to the cars. When this method of producing formaldehyde was perfected, the investigators sent to the Pullman Car Company and asked them for every kind of cloth which was used in the manufacture of their cars. They sent on some two or three hundred different patterns and colors of cloth, silks, satins, brocades, and the various cloths which they used, and the formaldehyde had no effect whatever upon them, except in two instances, where it affected the colors. I think one was a shade of blue and the other a shade of brown. The fabrics were not injured in the least. This formaldehyde will produce an odor, but by placing a given quantity of ammonia in the room the air there will be almost as pure as ozone within an hour or two.

The introduction of this method of disinfection is certainly the beginning of the happy era for the inspector of the boards of health whose duty it is to fumigate houses after the recovery or removal of cases of diphtheria and other diseases. He used to be looked upon as the *bête noire* of every one he came to visit. Everything about the house was turned topsy-turvy, while many utensils and ornamental articles were completely ruined. We are not obliged now to take our clothing to the steam sterilizer, for the inspectors know when they have killed every germ in the room. For instance, in testing, their method is to take a piece of cloth and saturate it with a bouillon solution of a culture of some kind of bacteria. That is wrapped up in several thicknesses of cloth and placed between the mattresses of a bed; the inspector then goes through the usual process, and on his return he removes the package containing the bacteria culture, which is sent to the board of health, and a microscopical examination shows them to be dead.

*Chairman Cooke.*—How much expense and how much time does this process require in the case of the Pullman cars?

*Dr. Swarts.*—The expense would not be over sixty or seventy cents for a car, and the labor would be that of a porter. It should be allowed to continue for, say, ten hours, although we know we can destroy them in four to five hours, and if a car was obliged to start out again in that time it would be in good condition. It is the custom to shut it up over night, and there is then no doubt that it is absolutely sterile.

*Dr. Bradley.*—I would like to ask if, as this remedy has been shown to be so destructive to bacteria, some preparation of it could not be used in the treatment of the disease itself? If it will prevent the germination of the bacilli and destroy them, could it not be used in some form to help the patient to resist or overcome the disease, the same as the antitoxin?

*Dr. Swarts.*—I have thought a great deal on that subject, and several persons are still working upon it. The trouble we meet with is that the treatment necessary to kill bacteria is injurious to tissue.

*Dr. Bradley.*—Is a patient who has once been affected by the disease, diphtheria, more subject to it than another person? My own son, when he was three or four years old, had quite a severe attack, and after he was pronounced fully recovered he had another slight attack. I never understood whether the second attack was one of those purely accidental cases or whether the fact of his having had the disease before rendered him more subject to it.

*Dr. Swarts.*—It does not, so far as we know, except it may reduce the general constitution to a point from which the patient may never recover.

*Dr. Bradley.*—How long is the immunity of the average person after recovery from a case of diphtheria?

*Dr. Swarts.*—That is a question which cannot be satisfactorily answered. We cannot say, as in typhoid, that it may continue for life. It is generally believed to be six months to a year, though cases have been known where persons have contracted diphtheria within four months from the time of their recovery from a previous attack. So, you see, it does not come under the head of those diseases which the old ladies claim it is better to have and be over with. You know, when they used to hear of a case of measles or mumps, they used to say, "It's a good thing for the children to have; let's go and get it." It would not be wise for a person to deliberately contract diphtheria.

*Chairman Cooke.*—Take it in this patient that you spoke of who went from May to October. I do not think you stated whether the case was fatal.

*Dr. Swarts.*—Oh, no, the patient was immunized. The organisms could not overcome the antitoxin quality in her blood, and, at the same time, in this case the organisms refused to be expelled from her system. As far as she was concerned, they could do her no harm. She was perfectly free from the disease, and could have gone about wherever she chose, and thereby would have been a greater source of danger than a person who was seriously ill with the disease, but who was confined to one room with only a doctor and a nurse.

*Dr. Clapp.*—How long after the infection is antitoxin available?

*Dr. Swarts.*—Before the nervous system has become saturated with the toxin of the diphtheritic germ. It is wholly a matter of the strength of the patient's system to resist the effects of this toxin. If the disease is brought to our attention in its early stages and we introduce the antitoxin, then we assist the system in its efforts, and thereby go on to the extent of immunizing the patient.

*Dr. Clapp.*—Then there is always hope from the effect of this unless the patient is very much depleted?

*Dr. Swarts.*—Unless the toxin has become distributed through the system to such an extent that it is beyond the power of the system, aided by the antitoxin, to overcome it. The horses which are used for the manufacture of antitoxin will live for an indefinite period if they do not succumb to the toxin during the process of immunization, a process which, as I have stated, extends sometimes over a period of eight months. They then proceed to draw from the horse, at the same time continuing the injection of the toxin, thereby keeping up the strength of the antitoxin quality of the blood to the standard as measured by its effect on guinea-pigs of certain weight. Some horses will not yield serum up to the standard strength, and when they find that a horse refuses to do so they sell him and get another horse. Occasionally they find one that is remarkable for the amount and quality of the serum produced. There is one old white horse in New York which produces great quantities of serum of excellent quality.

*Chairman Cooke.*—They milk them, same as you would a cow?

*Dr. Swarts.*—It has the same effect, but you get at it by a different process. The process is not a very painful one for the horses; they do not mind it.

*Chairman Cooke.*—Where are the most reliable preparations made?

*Dr. Swarts.*—Antitoxin is now made in several places in this country. Perhaps Mulford & Co., in Philadelphia, have paid the most attention to its manufacture. They are a druggist firm, and took up this question of antitoxin as soon as it came out, and are carrying on a perfectly clean business in every way. It is made for the city of Boston somewhere in the environs of Boston,—I do not remember now just where,—and by the State Board of Health of Massachusetts. The examinations of cultures sent in by physicians are made by the State, free of expense; the antitoxin is also given free to those patients who cannot afford it. At the office of the board of health we keep the antitoxin and syringes always on hand, and our instructions to physicians are, when they find a case of diphtheria, where the patient is too poor to buy what is necessary, to send at once to us and borrow the syringes and get a sufficient quantity of antitoxin, and use it quickly.

*Chairman Cooke.*—Any special method of cleaning those syringes after they have been used?

*Dr. Swarts.*—No; five per cent. carbolic acid will be sufficient. There would not be so much danger from the blood of the patient on the needle as from dirt which might be on the patient's skin.

I have brought down with me to-night a stained specimen of the Klebs-Löffler bacillus, which I will place in the microscope for your examination.

*Dr. Eames.*—I wish to express my appreciation of the manner in which the subject has been handled. I have been quite familiar with these methods and know something of the work which has been done in Providence, Rhode Island, from a friend of mine in that city, and I can say truthfully that I have been pleased and agreeably surprised to find my attention held and my ideas thus stimulated. I think we all must now have a better understanding of this subject.

The mention of individual communion-cups interested me, for the idea was original with myself and resulted in the first use of them in New England. I believe that the discussion of this question will show that diseases may be transmitted from sources which formerly we had no idea were means of transmitting and propagating disease, and that the study of bacteriology will enlighten us still further in regard to this matter. I am surprised that men in the medical profession still avow that the common communion-cup is not a source of danger.

Another point which should be emphasized is the fact that there are two forms of stomatitis which may be confounded with diphtheria,—the aphthous form, also that form which is produced by means of parasites, stomatitis mycosa. In both of these we have a white patch, especially in the early stages of the aphthous form, and it should be pointed out that in aphthous stomatitis the white spots appear on the inner surfaces of the lips, on the gums, and on the edges of the tongue, while in diphtheria, as has been pointed out, the white patches are found on the tonsils and on the edges of the palate, extending from behind forward. The tough, leathery, diphtheritic patch is in contrast to the softer consistency of the aphthous patch, which is easily detachable. The sore throat and more profound systemic disturbances of diphtheria are not present in stomatitis, as a rule.

*Dr. Werner.*—I, for one, appreciate the scientific, concise, non-repeating talk of this evening, and I am very glad I came. It is a pleasure to listen to one who can talk directly to a subject. Dr. Swarts has that faculty to an extent which I have seldom heard in speakers. It is something we all ought to learn; we have certainly had a profitable lesson in the address to-night. The method of disinfecting with wood alcohol and asbestos is of value to know.

Dr. Eames and others have spoken of communion-cups. Just compare for a moment the number of people who go to communion with the number of school children who drink in common from dirty cups. We ought all to support in an effective way any movement to improve the conditions existing in our school-houses; we ought to attend meetings which give expression to the care of textbooks, drinking-cups, pencils, etc., for the school is a decidedly more important place to begin our reform than the church.

*Dr. Potter.*—I cannot help saying a word about what has been something of a hobby with me for a good many years. It is undoubtedly a wise thing to agitate this matter of communion-cups and common drinking-cups, but we must not overlook our own instruments and the apparatus we are constantly using. I do not wish to go into particulars, but simply to urge the importance of knowing that our instruments are sterilized by a reliable process after every use.

*Dr. Swarts.*—That point—that the instrument may be a means of infection—I did not see fit to dwell upon, as I supposed that you had heard a great deal about it. I am glad, however, that it has been brought up, as it is right in line with something that I did intend to speak of.

In the patients coming to you for dental operations, should you see a throat that is intensely inflamed, with little dots on the tonsils, or any extended inflammation of the mucous membrane, it would be policy to discontinue work on that patient, for two reasons: first, you are running the risk of contracting diphtheria through the patient coughing in your face or infecting yourself by the handling of the patient; in the second place, the patient should be at home and receiving medical treatment, not only for his own sake, but for the good of the public; and by advising him in this matter and notifying the board of health, with the consent of the patient, you will be doing both the patient and the health department a service. I say "with the consent of the patient," for if you do not obtain his consent you are likely to get into trouble, or, at least, forfeit his friendship. I, unfortunately, as a physician, was called into a house where there had been a death in the family from diphtheria to make examinations of the remaining members. I told the lady that if I was to act in the capacity of physician I should be obliged to follow the procedures prescribed by law. She insisted upon my being the physician, and though only one of the family was really clinically sick, yet examination of the cultures in that family of four showed the Klebs-Löffler bacillus present in two persons. I followed the legal requirements, and three hours afterwards was dishonorably discharged. As a physician, I reported to the health department the examinations made, accompanied by the cultures which I obtained; as a bacteriologist, I reported to the health department the discovery of the Klebs-Löffler bacillus. The superintendent of the city health department ordered the patients quarantined and the house placarded in the usual manner. Another physician was called in, who declared that the patients were entirely free from diphtheria, and as a result there is now a five-thousand-dollar lawsuit hanging over my head for malpractice. So get the consent of the patient before you take any steps in a matter of this kind; but it would be a great help to the health department in this city, and in all cities, if cases of this kind were looked after at the earliest moment possible, because certain isolated cases might be the means of transmitting the disease to such an extent as to cause an epidemic.

*Dr. Clapp.*—To what extent is the quarantine carried?

*Dr. Swarts.*—Our practice in Providence is to quarantine the patient with one attendant, and, so far as practicable, to confine them to not more than two rooms. Of course, it is often difficult to do this, especially when your case is in a tenement house of, say, a half-dozen rooms, and there are seven children and three adults living there. We then insist that no children in that family shall attend school or Sunday-school, and that a watch be kept over them to prevent them from playing with other children. The father of the family is allowed to continue at work, and he also is instructed as to his relations with his family and with other people. The proper thing to do in a case of diphtheria would be the quarantining of every one in the house, if necessary, feeding the family at the public expense, and paying the bread-winner his customary wages, and I think that eventually this will be done. It is going to be expensive, but when compared with the cost of treating the cases it is more economical.

WILLIAM H. POTTER, D.M.D.,
*Editor American Academy of Dental Science.*

